# Prognostic Role of Common MicroRNA Polymorphisms in Cancers: Evidence from a Meta-Analysis

**DOI:** 10.1371/journal.pone.0106799

**Published:** 2014-10-22

**Authors:** Lingzi Xia, Yangwu Ren, Xue Fang, Zhihua Yin, Xuelian Li, Wei Wu, Peng Guan, Baosen Zhou

**Affiliations:** 1 Department of Epidemiology, School of Public Health, China Medical University, Shenyang, Liaoning Province, China; 2 Key Laboratory of Cancer Etiology and Intervention, University of Liaoning Province, Shenyang, China; University of North Carolina School of Medicine, United States of America

## Abstract

**Background:**

The morbidity and mortality of cancer increase remarkably every year. It's a heavy burden for family and society. The detection of prognostic biomarkers can help to improve the theraputic effect and prolong the lifetime of patients. microRNAs have an influential role in cancer prognosis. The results of articles discussing the relationship between microRNA polymorphisms and cancer prognosis are inconsistent.

**Methods:**

We conduct a meta-analysis of 19 publications concerning the association of four common polymorphisms, mir-146a rs2910164, mir-149 rs2292832, mir-196a2 rs11614913 and mir-499 rs3746444, with cancer prognosis. Pooled Hazard Ratios with 95% Confidence Intervals for the relationship between four genetic polymorphisms and Overall Survival, Recurrence-free Survival, Disease-free survival, recurrence are calculated. Subgroup analysis by population and type of tumor are conducted.

**Results:**

GG genotype of mir-146a may be the protective factor for overall survival, especially in Caucasian population. C-containing genotypes of mir-196a2 act as a risk role for overall survival. The same result exists in Asian population, in Non-Small Cell Lung Cancer and digestive cancer. The patients with C allele of mir-149 have a better overall survival, especially in Non-Small Cell Lung Cancer. No significant results are obtained for mir-499 polymorphisms.

**Conclusions:**

Genetic polymorphisms in mir-146a, mir-196a2 and mir-149 may be associated with overall survival. This effect varies with different types of cancer. Genetic polymorphism in mir-499 may have nothing to do with cancer prognosis.

## Introduction

Cancer is a primary cause of morbidity and mortality in the vast marjority of regions in the world. The world estimated incidence and mortality in 2012 is 14.09 million and 8.2 million, respectively [1], [2]. According to the development trend, the new cases in 2030 will reach 22.2 million [3]. Cancer itself and medical treatment for cancer have been a heavy burden for both family and society. A constantly increased attention, these years, focuses on the disclosure of the methods that could treat patients effectively and economically. The detection of biomarkers will help to diagnose underlying patients at an early period and the identification of targeted genetic sites can promote the theraputic effect and prolong the lifetime of patients.

With the development in medical researches, it is widely recognized that the polymorphisms in microRNA genes can act as an essential role in carcinogenesis and progression. microRNAs (miRNAs) are the endogenous, small non-coding RNAs with a length of 18–25 nucleotides. The seed region of the miRNAs can recognize and complementarily combine with the 3′UTR of the specified mRNA, thus disrupting the biosynthesis. Numerous studies have detected a higher or lower level of microRNAs in patients with poor outcome than those with good outcome [4], [5]. The polymorphisms in microRNAs may alter their ability to combine with the targeted mRNA and consequently strenghen or weaken their ability to disrupt biosynthesis [6].

Once detected, microRNA have attached much attention for its multiple roles in tumorigenesis.

miR-146a shows a more extensive role in cancer. It may target to TRAF6 [7], IRAK1 [8], and thus play an influential role in the prognosis of patients suffering inflammation after surgery or chemoradiotherapy. It can also up-regulate the expression of PDGFRA [9] to enable the regeneration capacity of endothelial cells. Two peaks of miR-146a appear in 8 h and 24 h after chemoradiotherapy in the study [10]. It can influence the expression of WASF2. WASF2 is the downstream molecular that can transmit the GTPase signal to actin skeletal, thus affect the ability to migrate [11].

Others find miR-196 family can target to HOXC8 [12] and LSP1. And the polymorphism of the gene can alter the ability [6]. HOXC may influence the ability of migration and invasian of cells and the ability depends on the ratio of the expression between mir-196a and HOXC8 mRNA [12]. The high expression of LSP1 in multiple myeloma can influence the effect of a new anticancer drug, Bortezomib, on inducing cell apoptosis.

Studies determined the direct role of miR-149 in the Forkhead Box M1(FOXM1) mRNA to prevent the EMT process, which is important in proliferation of tumor [13]. Expression of mir-149 may affect the Puma maturation to prolong the lifetime of cells [14]. In gastric cancer, miR-149 can prevent the cell cycle by down-regulating ZBTB2 protein in ARF-HDM2-p53-p21 pathway [15]. miR-149 also can induce cell apotosis by down-regulating the expression of Akt1, E2F1 and b-Myb [16], [17].

The underlying biological mechanism of mir-499 in cancer is not elucidated. Some bioinformatic tools are used to explore the potential mechanism. Two breast cancer suppressors, NBN and BCL2L14, are predicted targets of hsa-miR-499 [18].

Genetic polymorphisms in mirnas may influence the cancer prognosis either by affecting the maturation [6], [19], [20] or by altering ability to combine with target mRNAs [6]. Studies showed that SNP in mir-146a can influence the expression of mature miR-146a [20], [21]. Mir-196a2 polymorphism was observed to alter the ability to combine with target [6]. Mir-149 polymorphism can affect its ability to regulate downstream targets by affecting the maturation of miR-149 [22].

Recently, the emerging role of microRNA polymorphisms in prognosis of cancer patients attracts some interest. In different types of cancers, microRNAs show to have different roles. In glioma [23], miRNAs show the risk role for deaths, while in gastric cancer [24], they may function as a protective factor for overall survival. Although in the same type of cancer, microRNA may have different functions. This may result from the small sample size in a single study. With the controversial results, we conduct this meta-analysis to evaluate the relationship between common genetic polymorphisms in four microRNAs (mir-146a rs2910164, mir-149 2292832, mir-196a2 rs11614913, mir-499 rs3746444) with cancer prognosis. To the best of our knowledge, this is the first meta-analysis concerning the four genetic polymorphisms with cancer prognosis.

## Methods

### Search strategy

This meta-analysis was carried out in accordance with the guidelines of the meta-analysis of the Observational Studies in Epidemiology group (MOOSE) [25]. We took a comprehensive search strategy in this study. The search strategy used the following terms variably combined by “microRNA”, “mir”, “cancer”, “carcinoma”, “tumor”, “survival”, “overall survival”, “Recurrence”, “disease-free survival”, “recurrence-free survival”, “disease-specific survival”, “prognosis” and “prognostic”. All of the avaliable database or online sources, such as PubMed, Scie, CBM, google scholar, CNKI, WanFang, were searched; After a browse of the title and abstract, the articles, including conference abstract, original articles and reviews, were screened out; The reference lists were searched as well. The last time for search on March, 2014. Only reviews published in English were evaluated.

Eligible studies included in this meta-analysis met the following criteria: (i) Discuss the role of the four microRNA polymorphisms in cancer; (ii) Investigate the overall survival outcome or other clinical variables, such as RFS, DSS, DFS and recurrence; (iii)HR and 95%CI are accessable. Articles were excluded based on any of the following criteria: (i)Duplicated articles or data; (ii) Lack of HR and 95%CI.

### Data extraction

Two authors independently extracted data. If not consistent, the third author will join in to discuss. Any controvery will be solved by voting. All the data were subject to consensus. We contacted the authors of the articles for missing data by email. We extracted information including first author's name, year of publication, origin of the study population, size of the study population, type of tumor, genotyping method, the polymorphism site, method of survival analysis, HR(95%CI), and the follow-up time(months). HR values>1 were considered indicative of significant associations with poor outcome.

### Statistical methods

Heterogeneity was assessed using Q statistics (P<0.05 was considered heterogeneous). Any significant heterogeneity among the studies was resolved using the random-effects model. Otherwise, the fixed-effects model was used. The I^2^ statistic, which measures the percentage of the total variation across studies that is due to heterogeneity rather than to chance, was also assessed. The effect of miRNA polymorphisms on survival outcome (OS) were estimated using forest plots. Stratified analysis of pooled HR and 95%CI for the relationship between polymorphisms with cancer prognosis in various population and cancers was done. Pooled HR was calculated using a fixed-effects model or random-effects model as appropriate. Pooled HR>1 indicated poor prognosis and was considered statistically significant if the 95% CI did not contain 1 [26]. Bonferroni correction was applied to control the potential false positive error. In this meta-analysis, the multiple comparision for mir-146a, mir-196a2, mir-149 and mir-499 was performed 13, 12, 9 and 9 times, respectively. The statistically significant P-value after correction for mir-146a, mir-196a2, mir-149 and mir-499 is 0.0038(0.05/13), 0.004(0.05/12), 0.0056(0.05/9) and 0.0056(0.05/9). Publication bias was evaluated using the funnel plot and Begg's test. P>0.05 was considered indicative of a lack of publication bias [27]. Sensitivity analysis was conducted by eliminating articles one by one. All analyses were performed using STATA vision 13.0. All of the P-value is two sided and a P-value less than 0.05 was considered to be statistically significant.

## Results

### Study Characteristics

**Figure 1:**
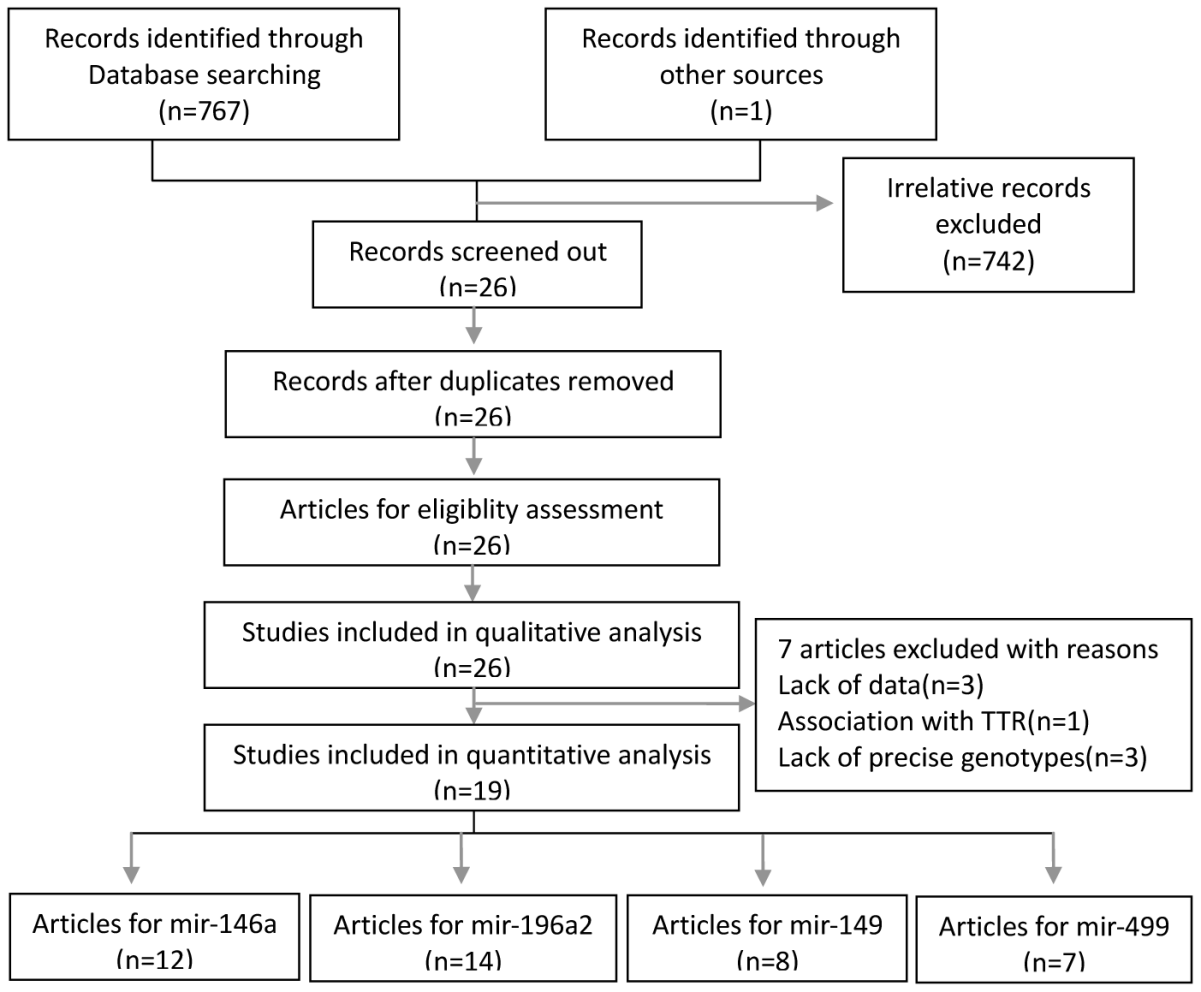
The flowchart of the selection process. We utilized a comprehensive searching strategy to screen out potential related articles as far as possible. 26 articles focusing on the association between the four genetic polymorphisms and cancer prognosis are screened out. 7 articles are excluded in quantitative ananlysis for lack of data to calculate pooled HR and 95%CI.

The flow diagram of the study selection process is presented in Figure 1. Nineteen [7], [23], [24], [28]–[43] eligible publications are included in this meta-analysis with 8890 patients totally. Seven [44]–[50] are excluded for lack of data and precise genotypes. These eligible articles were published from 2008 to 2014. Twelve [23], [24], [28]–[35], [37], [43] studies concerning the relationship between mir-146a polymorphism and cancer prognosis. Of them, nine articles focus on the relationship with overall survival, one on the relationship with recurrence, three on relationship with recurrence-free survival(RFS) and three on relationship with disease-free survival(DFS). The number of the articles concerning the relationship between polymorphisms in mir-196a2, mir-149 and mir-499 and cancer prognosis is respectively fourteen [7], [24], [29]–[31], [33]–[35], [37]–[41], [43], eight [24], [29], [31], [33], [34], [36], [37], [42] and seven [24], [29]–[31], [33], [34], [37]. The original population contain American, Korean, Chinese, Indian, Spainish and German. The type of tumor covers colorectal cancer (CRC), gastric cancer (GC), non-small cell lung cancer (NSCLC), esophageal squamous cell carcinoma (ESCC), hepatocellular carcinoma (HCC), bladder cancer, squamous cell carcinoma of prostate (SCCOP), head and neck squamous cell carcinoma (HNSCC), hodgkin lymphomam, nasopharyngeal and malignant lymphoma. Characteristics of eligible articles are summarized in Table S1. The original data for this meta-analysis are listed in Table S2.

### Main meta-analysis results

**Figure 2:**
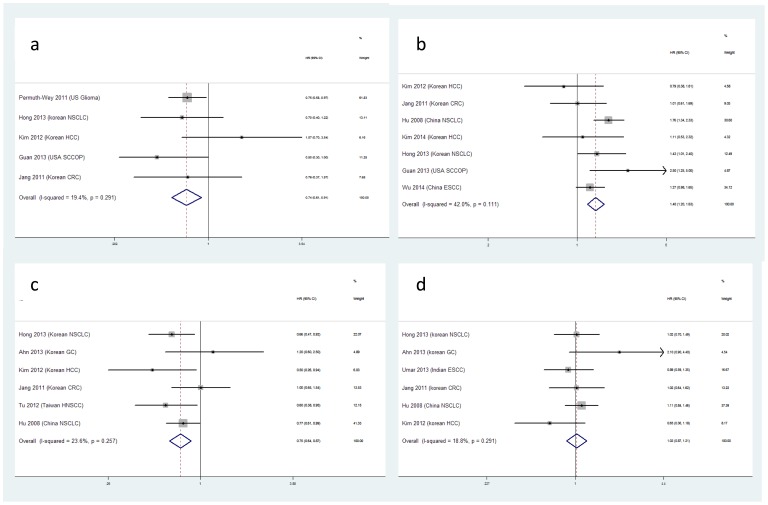
The main results of meta-analysis for the four genetic polymorphisms. The forest plots for pooled HR and 95%CI estimated to demonstrate the role of mir-146a in Dominant model(a), mir-196a2 in Recessive model(b), mir-149 in Dominant model(c) and mir-499 in AG vs AA(d) in overall survival.

**Figure 3:**
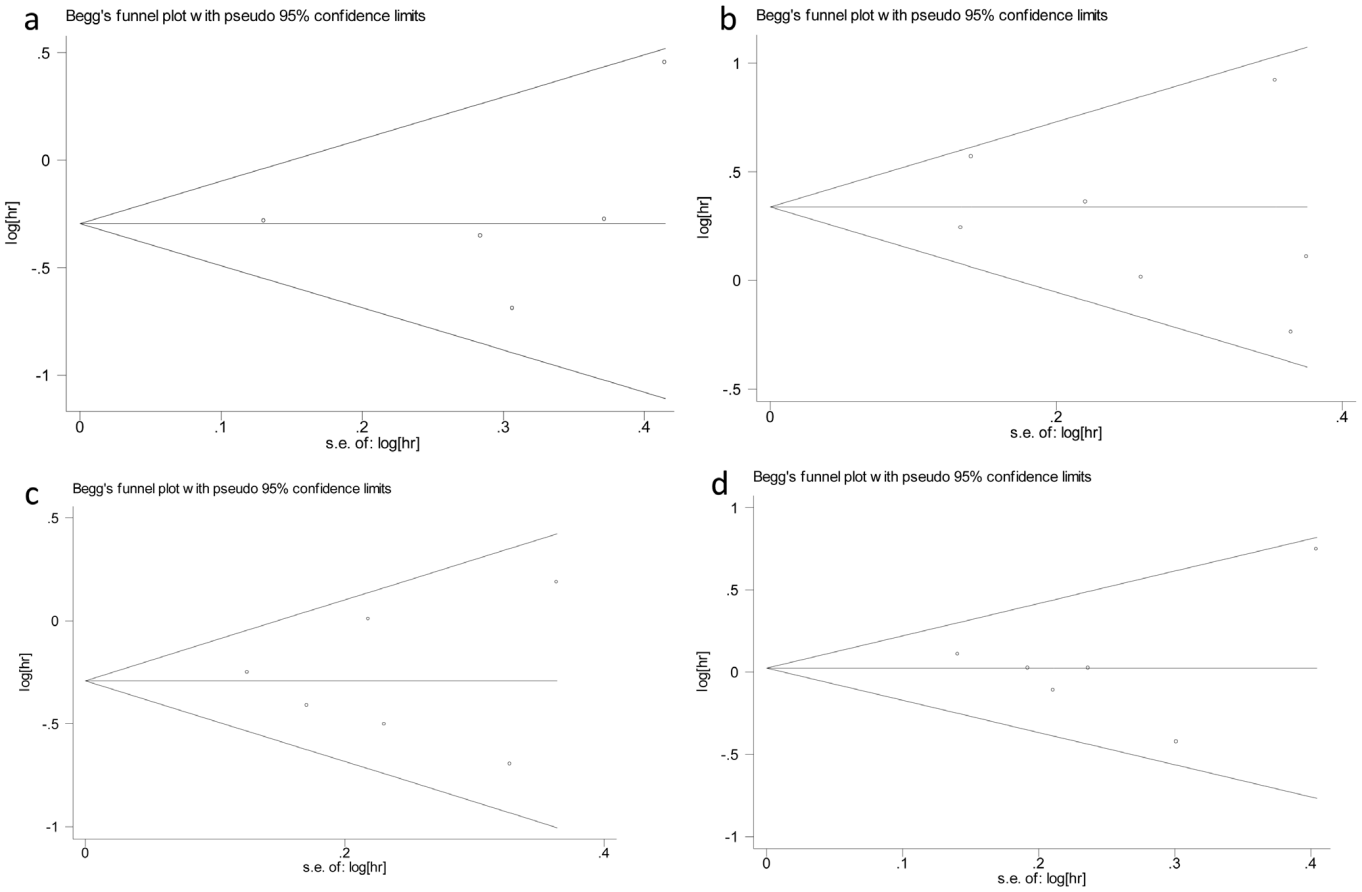
Funnel plots for the four genetic polymorphisms. Funnel plots of the publication bias for mir-146a in Dominant model(a), mir-196a2 in Recessive model(b), mir-149 in Dominant model(c) and mir-499 in AG vs AA(d).

The meta-analysis results for relationship between polymorphisms and cancer overall survival are summarized in Table 1. The forest plot and funnel plot are listed in Figure 2 and Figure 3. The results of subgroup analysis by original population are summarized in Table 2 and the results of subgroup analysis by type of tumor are summarized in Table 3.

**Table 1:** Pooled HRs and 95%CIs from meta-analysis for OS.

Snp(rs)	No. of studies	No. of patients	Model	HR(95%CI)	P-value	Heterogeneity (I^2^, P-value)
Mir-146a rs2910164	8	2906	GG vs CC	1.088(0.921–1.286)	0.319	18.3%, 0.286
	5	2046	CG vs CC	0.938(0.768–1.145)	0.527	38.9%, 0.162
	5	1560	DOM	0.74(0.61–0.91)	0.004	19.4%, 0.291
Mir-196a2 rs11614913	7	2577	CC vs TT	1.129(0.757–1.683)	0.552	73.3%, 0.001
	3	1027	CT vs TT	1.710(1.070–2.735)	0.025	23.4%, 0.271
	7	2401	DOM	1.148(0.881–1.494)	0.307	67.5%, 0.002
	6	1940	REC	1.401(1.203–1.633)	<0.001	42.0%, 0.111
Mir-149 rs2292832	6	2046	CC vs TT	0.81(0.615–1.065)	0.131	37.3%, 0.172
	4	1383	CT vs TT	0.748(0.585–0.955)	0.020	0.0%, 0.432
	6	2319	DOM	0.747(0.638–0.875)	<0.001	23.6%, 0.257
	3	875	REC	0.678(0.425–1.083)	0.104	36.5%, 0.207
Mir-499 rs3746444	5	2040	GG vs AA	0.971(0.620–1.520)	0.897	0.0%, 0.771
	6	2199	AG vs AA	1.025(0.866–1.214)	0.733	18.8%, 0.291
	3	1177	DOM	1.104(0.787–1.549)	0.568	0.0%, 0.661

*DOM: dominant model, REC:recessive model.

**Table 2:** Stratified analysis by group for different population OS.

Snp(rs)	population	No. of studies	No. of patients	Model	HR(95%CI)	P-value
Mir-146a rs2910164	Asian	6	2123	GG vs CC	1.073(0.896–1.286)	0.444
	Others**	2	482	GG vs CC	1.179(0.768–1.810)	0.451
	Asian	5	1745	CG vs CC	0.938(0.768,1.145)	0.527
	Asian	3	922	DOM	0.861(0.583–1.271)	0.451
	American	2	638	DOM	0.706(0.558–0.894)	0.004
Mir-196a2 rs11614913	Asian	6	1917	DOM	1.061(0.977–1.153)	0.161
	Asian	5	2046	CC vs TT	1.086(0.901–1.310)	0.387
	Asian	6	2689	REC	1.361(1.163–1.592)	<0.001
Mir-499 rs3746444	Asian	5	2304	AG vs AA	1.055(0.876–1.269)	0.573
	Asian	4	1887	GG vs AA	1.041(0.607–1.783)	0.885

*DOM: dominant model, REC:recessive model.

**The others include American and Indian population.

**Table 3:** Stratified analysis by type of tumor for OS.

SNP(rs)	Type of tumor	No. of study	No. of patients	Model	HR(95%CI)	P-value
rs2910164	Digestive cancer	5	1558	GG vs CC	1.116(0.897–1.388)	0.325
		3	1027	CG vs CC	0.884(0.628–1.244)	0.479
		3	895	DOM	0.752(0.502–1.125)	0.166
	NSCLC	3	1348	GG vs CC	1.051(0.812–1.361)	0.704
		2	1019	CG vs CC	0.967(0.756–1.236)	0.787
rs11614913	Digestive cancer	5	1558	CC vs TT	0.779(0.610–0.996)	0.046
		6	1917	DOM	1.061(0.977–1.153)	0.161
		5	1670	REC	1.235(1.008–1.512)	<0.001
	NSCLC	2	1020	CC vs TT	1.642(1.244–2.165)	<0.001
		2	1019	REC	1.657(1.312–2.092)	<0.001
rs2292832	Digestive cancer	3	1027	CC vs TT	0.892(0.519–1.533)	0.679
		3	1027	CT vs TT	0.835(0.597–1.167)	0.291
		3	1027	DOM	0.875(0.636–1.204)	0.411
	NSCLC	2	1019	CC vs TT	0.725(0.519–1.012)	0.058
		2	1019	DOM	0.733(0.601–0.893)	0.002
rs3746444	Digestive cancer	3	1021	GG vs AA	1.004(0.535–1.887)	0.989
		4	1180	AG vs AA	0.958(0.740–1.242)	0.748
	NSCLC	2	1019	GG vs AA	0.938(0.496–1.775)	0.844
		2	1019	AG vs AA	1.078(0.862–1.347)	0.511

*DOM: dominant model, REC:recessive model.

### Mir-146a

In this study, we set dominant model of mir-146a as GG vs CC+CG, recessive model CC vs GG+CG. A significant result existing in dominant model indicats the protective role of homologous frequent genotype in overall survival (HR = 0.74, 95%CI 0.61–0.94, P = 0.004, Table 1). When stratified, the association between mir-146a polymorphisms and overall survival was observed in American population in dominant model (P = 0.004, Table 2). No significant association between mir-146a polymorphism and digestive cancer or NSCLC was observed in our study(Table 3). While, in Wang et al. [32] and Lin et al. studies [49], Mir-146a polymorphisms may be associated with lung cancer recurrence, moreover the polymorphisms may be related with DFS (for GG vs CC+CG, HR = 0.649, 95%CI 0.423–0.996, Table S3). We observe no association with RFS(HR = 0.669, 95%CI 0.371–1.205, Table S3).

### Mir-196a2

Here, we set dominant model as CC+CT vs TT, recessive model CC vs CT+TT. CT genotype of mir-196a2 have a significantly risk role in overall survival (HR = 1.710, 95%CI 1.070–2.735, P = 0.025, Table 1). However, the association was greatly weakened after Bonferroni correction (P>0.004). Even so, a robust association was observed between CC genotype and poor overall survival in recessive model (HR = 1.401, 95%CI 1.202–1.633, P<0.001, Table 1). Consistently, the robust association was observed in Asian population (HR = 1.361, 95%CI 1.163–1.592, P<0.001, Table 2) and in digestive cancer (HR = 1.235, 95%CI 1.008–1.512, P<0.001, Table 3) and NSCLC (HR = 1.657, 95CI 1.312–2.092, P<0.001, Table 3). Moreover, C allele containing genotypes may be associated with RFS (for CT vs TT, HR = 0.675, 95%CI 0.485–0.94; for CC+CT vs TT, HR = 0.687, 95%CI 0.504–0.936, Table S3). No association with DFS (Table S3) was observed in this meta-analysis.

### Mir-149

For mir-149, we set dominant model as CC+CT vs TT, recessive model CC vs CT+TT. In our study, we observe the protective role of C allele in cancer overall survival and a trend in the relationship with the number of C allele(for CC vs TT, HR = 0.81, 95%CI 0.615–1.065, P = 0.131; for CT vs TT, HR = 0.748, 95%CI 0.585–0.955, P = 0.020; for dominant model, HR = 0.747, 95%CI 0.638–0.875, P<0.001, Table 1). No significant association was observed between rs11614913 and digestive cancer overall survival in any model (Table 3). While, the genetic variant may be significantly associated with NSCLC(for CC vs TT, HR = 0.725, 95%CI 0.519–1.012, P = 0.058; for dominant model, HR = 0.733, 95%CI 0.601–0.893, P = 0.002, Table 3).

### Mir-499

We set dominant model as AG+GG vs AA for mir-499 polymorphism. In our meta-analysis, we didn't gain any significant results in any model (Table 1). Results from stratified analysis indicated that mir-499 polymorphism may have no association with cancer overall survival in Asian population (Table 2). No significant association was observed between rs3746444 and digestive cancer overall survival or NSCLC in any model (Table 3).

## Discussion

In this meta-analysis, we find that GG genotype of mir-146a may be a protective factor for OS, especially in Asian population. Although the statistically significant association with recurrence and DFS was detected in our study, we should notice that there are only two articles included. Nonetheless, the results imply the role of mir-146a in cancer prognosis and we should lucubrate in the future. For mir-196a2, we find an interesting matter. The C allele is a risk factor for overall survival, whereas it is a protective factor for RFS. This may result from the different types of cancers, various follow-up time period or the differences in baseline characteristics. A notable thing is that the association between mir-196a2 polymorphism and RFS is not consistent with the report in Chae [46]'s article. In Chae's article [46], a P-value larger than 0.05 is reported for the relationship between them. The article [46] is not included in this meta for it doesn't provide HR and 95%CI. This meta-analysis implies that the C allele of mir-149 may have a protective role in cancer prognosis. No statistically significant results were concluded for mir-499 polymorphisms. This may result from a relatively small number of articles discussing the association of mir-499 polymorphisms with cancer prognosis. Stratified analysis implies the association of the polymorphisms in mir-196a2 and mir-149 with NSCLC, while the association of the four polymorphisms with digestive cancer overall survival was only observed in mir-196a2 polymorphisms in this meta-analysis.

We conducted the stratified analysis by population to determine the association of these four microRNA polymorphisms with cancer prognosis. For the articles in hand, we observe that most of the studies are conducted in Asian population. Only 4, 2 and 1 are conducted respectively in Caucasion, European and Indian population. The stratified analysis by type of cancer is conducted. With a small number of articles included, the number of articles for each subgroup is 5 to the most. What a pity that we are not able to conduct stratified analysis by age, gender, somking status or other pathologic stages for insufficient articles. Some studies have reported the significant role for these polymorphisms when subgrouped by age [7], [23], [44], gender [23], [29], [44], or other pathologic stages [7], [29].

Some defects exists in our meta-analysis. Firstly, the number of articles included is relatively small, especially for mir-149 and mir-499. Secondly, we conduct stratified analysis by population, most of which are Asian, and type of tumor, most of which are digestive cancer and NSCLC, rather than other baseline characteristics. Thirdly, some heterogeneity exist in the relationship between mir-196a2 polymorphism and cancer prognosis. When exclude the Wang's article [7], the heterogeneity disapear. This may result from the different role of the polymorphism in cancer prognosis. For mir-196a2 polymorphism in Wang's article, the CC genotype is a protective factor(HR = 0.72, 0.55–0.95) in gastric cancer. Excluded, the pooled HR equals 1.476 and 95%CI ranges between 1.222–1.782 which imply the risk factor for mir-196a2 polymorphism in all cancers. This may also result from the difference in baseline characteristics. Fourthly, a P-value of 0.04 for publication bias is obtained in the association between mir-149 polymorphism and overall survival in cancers in dominant model. This may result from the small number of articles included in this meta-analysis.

Nonetheless, many advantages exist in our meta-analysis. First of all, this is the first meta-analysis concerning the relationship between the four common polymorphisms in microRNA and cancer prognosis. What's more, no heterogeneity exists in the models for the polymorphisms in mir-146a and mir-499. No publication bias is observed in the models for the polymorphisms in mir-146a, mir-149 and mir-499. Consequently, the results in our meta-analysis are stable and reliable. The last but not the least, the total number of patients in our meta-analysis is relatively large, which reaches 8057 totally.

## Conclusions

All of the results observed in our meta-analysis support the role of polymorphisms in mir-146a, mir-149 and mir-196a2 in cancer prognosis, with their functions may differ from population to population, from one type of cancer to another. More studies with a larger sample size in different population are needed to determinate the role in various cancers.

## Supporting Information

Table S1 **Basic information of the articles included in the meta-analysis.**
(DOC)

Table S2 **The original data for the meta-analysis.**
(DOC)

Table S3 **The association of mirna polymorphisms with DFS and RFS.**
(DOC)

File S1 **The seven excluded articles and the reasons.**
(DOC)

Checklist S1 **PRISMA checklist.**
(DOC)
