# Supplementary material for: Prognostic Role of Common MicroRNA Polymorphisms in Cancers: Evidence from a Meta-Analysis
Source: PLoS One. 2014 Oct 22;9(10):e106799. doi: 10.1371/journal.pone.0106799 (PMC4206268; doi:10.1371/journal.pone.0106799)
Supplement: Table S1 — Basic information of the articles included in the meta-analysis. (DOC) [file pone.0106799.s001.doc]

Table S1 Basic information of the articles included in the meta-analysis

| Author(yr) | Country | No. | Type of Tumor | Genotyping/DNA sample | mir | Survival analysis | Follow-up (months) | Mean/median  age(SD/range) | Male (%) | Stage |
| --- | --- | --- | --- | --- | --- | --- | --- | --- | --- | --- |
| Hu(2008) | China | 663 | NSCLC | PCR-RFLP/venous blood | mir-146a  mir-149  mir-196a2  mir-499 | OS | NA | 60.17(9.65) | 73.9 | I-IV |
| Christensen(2010) | USA | 484 | HNSCC | Taqman/blood or buccal cell | mir-196a2 | OS | NA | 59.4(11.6) | 74.2 | I-IV |
| Permuth-Wey(2011) | US | 329 | glioma | GoldenGate/oral rinse and saliva | mir-146a | OS | Median11.5 | 55(19-89) | 61 | NA |
| Jang(2011) | Korea | 407 | CRC | PCR-RFLP/blood | mir-146a  mir-149  mir-196a2  mir-499 | OS,RFS | Median 41 | 61.4(12.4) | 55.8 | 0-IV |
| Kim(2012) | Korea | 159 | HCC | PCR-RFLP/whole blood | mir-146a  mir-149  mir-196a2  mir-499 | OS | Median | 56.06(11.02) | 76.7 | I-IV |
| Wang(2012) | China | 199 | Bladder | Taqman/venous blood | mir-146a | Recurrence | NA | 64.8(12.1) | 79.9 | G1-G3 |
| Yoon(2012) | Korea | 388 | NSCLC | Taqman/peripheral blood | mir-146a  mir-196a2 | RFS | Median 36.8 | 62.7(9.2) | 67 | IA-IIIA |
| Tu(2012) | Taiwan | 273 | HNSCC | PCR-RFLP/blood | mir-149 | OS | Mean 50.4 | 53.0(11) | 91.9 | I-IV |
| Chae(2013) | Korea | 343 | CRC | PCR-RFLP/tissue | mir-146a | RFS,DFS | median42.3 | 62.4(11.4) | 54.6 | I-IV |
| Hong(2013) | Korea | 356 | NSCLC | PCR-RFLP/NA | mir-149  mir-146a  mir-196a2  mir-499 | OS,DFS | NA | NA | 76.6 | I-IIIA |
| Ahn(2013) | Korea | 461 | GC | PCR-RFLP/leukocytes | mir-146a  mir-149  mir-196a2  mir-499 | OS | mean61.2 | 58.08(12.35) | 63.1 | I-IV |
| Umar(2013) | India | 153 | ESCC | PCR-RFLP/peripheral blood leukocytes | mir-196a2  mir-146a  mir-499 | OS | Median8.5 | 57.03(11.36) | 71.3 | NA |
| Guan(2013) | USA | 309 | SCCOP | PCR-RFLP/blood | mir-146a  mir-196a2  mir-149  mir-499 | OS,DSS,DFS | Median21.2 | 53(28-38) | 87.1 | I-IV |
| Liu(2013) | Taiwan | 95 | OSCC | PCR-RFLP/blood | mir-196a2 | OS | Median 32.5 | 54.1 | 91.4 | I-IV |
| Navarro(2013) | Spain | 141 | Hodgkin lymphoma | Taqman/tissue | mir-196a2 | DFS | Median 50 | 32(13-89) | 51.1 | I-IV |
| Wang(2013) | China | 749 | GC | Taqman/blood | mir-196a2 | OS | Median68.5 | NA | 73.1 | I-IV |
| Zhang(2013) | China | 98 | ML | PCR-RFLP/blood | mir-149 | OS | NA | NA | 46 | NA |
| Kim(2014) | Korea | 417 | HCC | Taqman/blood | mir-196a2 | OS | NA | 57.5(25-82) | 66.9 | NA |
| Wu(2014) | China | 378 | ESCC | PCR-LDR/venous blood | mir-146a  mir-149 | OS | Median 20 | 57.9(9.9) | 58.7 | III-IV |

*NA:the data not mentioned, OS: overall survival, RFS: recurrence-free survival, DFS:disease-free survival, DSS:disease-specific survival
