# Supplementary material for: Prognostic Role of Common MicroRNA Polymorphisms in Cancers: Evidence from a Meta-Analysis
Source: PLoS One. 2014 Oct 22;9(10):e106799. doi: 10.1371/journal.pone.0106799 (PMC4206268; doi:10.1371/journal.pone.0106799)
Supplement: Table S2 — The original data for the meta-analysis. (DOC) [file pone.0106799.s002.doc]

| Author(yr)  Table S2 The original data for the meta-analysis | SNP | Genotypes | No. | HR(95%CI) | | | |
| --- | --- | --- | --- | --- | --- | --- | --- |
|  |  |  |  | OS | RFS | DFS | recurrence |
| Hu(2008) | rs2910164 | CC | 122 | 1 |  |  |  |
|  |  | CG | 324 | 1.05(0.75-1.47) |  |  |  |
|  |  | GG | 217 | 1.28(0.89-1.82) |  |  |  |
|  | rs2292832 | GG | 76 | 1 |  |  |  |
|  |  | GT | 300 | 1.03(0.69-1.54) |  |  |  |
|  |  | TT | 287 | 1.32(0.89-1.97) |  |  |  |
|  |  | GG/GT | 376 | 1 |  |  |  |
|  |  | TT | 287 | 1.29(1.01-1.65) |  |  |  |
|  | rs11614913 | TT | 184 | 1 |  |  |  |
|  |  | CT | 316 | 1.09(0.81-1.46) |  |  |  |
|  |  | CC | 163 | 1..86(1.32-2.62) |  |  |  |
|  |  | TT/CT | 500 | 1 |  |  |  |
|  |  | CC | 163 | 1.76(1.34-2.33) |  |  |  |
|  | rs3746444 | AA | 491 | 1 |  |  |  |
|  |  | AG | 158 | 1.11(0.84-1.46) |  |  |  |
|  |  | GG | 14 | 1.24(0.55-2.83) |  |  |  |
| Christensen(2010) | rs11614913 | CC/CT | 182 | 1 |  |  |  |
|  |  | TT | 302 | 0.8(0.4-1.6) |  |  |  |
| Permuth-Wey(2011) | rs2910164 | GG | 194 | 1 |  |  |  |
|  |  | CG | 105 | 1.44(1.1-1.88) |  |  |  |
|  |  | CC | 30 | 0.99(0.61-1.60) |  |  |  |
|  |  | CG/CC | 135 | 1.33(1.03-1.72) |  |  |  |
| Jang(2011) | rs2910164 | CC | 137 | 1 | 1 |  |  |
|  |  | CG | 217 | 1.203(0.746-1.939) | 1.128(0.702-1.813) |  |  |
|  |  | GG | 53 | 0.854(0.384-1.899) | 1.494(0.752-2.97) |  |  |
|  |  | CG/GG | 270 | 1.141(0.715-1.822) | 1.186(0.751-1.874) |  |  |
|  |  | CC/CG | 354 | 1 | 1 |  |  |
|  |  | GG | 53 | 0.757(0.365-1.571) | 1.386(0.748-2.568) |  |  |
|  | rs2292832 | TT | 194 | 1 | 1 |  |  |
|  |  | CT | 168 | 1.008(0.64-1.588) | 1.201(0.759-1.9) |  |  |
|  |  | CC | 45 | 0.99(0.475-2.062) | 0.903(0.427-1.908) |  |  |
|  |  | CT/CC | 213 | 1.004(0.654-1.542) | 1.134(0.731-1.761) |  |  |
|  |  | TT/CT | 362 | 1 | 1 |  |  |
|  |  | CC | 45 | 0.986(0.487-1.995) | 0.822(0.405-1.665) |  |  |
|  | rs11614913 | TT | 111 | 1 | 1 |  |  |
|  |  | CT | 195 | 1.08(0.631-1.85) | 0.763(0.445-1.311) |  |  |
|  |  | CC | 101 | 1.07(0.574-1.993) | 0.998(0.555-1.794) |  |  |
|  |  | TC/CC | 296 | 1.077(0.649-1.788) | 0.845(0.514-1.387) |  |  |
|  |  | TT/CT | 306 | 1 | 1 |  |  |
|  |  | CC | 101 | 1.017(0.611-1.692) | 1.187(0.732-1.923) |  |  |
|  | rs3746444 | AA | 259 | 1 | 1 |  |  |
|  |  | AG | 137 | 1.02(0.641-1.624) | 0.939(0.576-1.531) |  |  |
|  |  | GG | 11 | 1.135(0.275-4.682) | 1.707(0.524-5.562) |  |  |
|  |  | AG/GG | 148 | 1.027(0.652-1.619) | 0.983(0.613-1.578) |  |  |
|  |  | AA/AG | 396 | 1 | 1.752(0.547-5.616) |  |  |
|  |  | GG | 11 | 1.027(0.276-4.6) |  |  |  |
| Kim(2012) | rs2910164 | CC | 20 | 1 |  |  |  |
|  |  | CG | 39 | 0.903(0.475-1.717) |  |  |  |
|  |  | GG | 8 | 1.456(0.568-3.729) |  |  |  |
|  |  | CG/GG | 47 | 0.964(0.517-1.797) |  |  |  |
|  |  | CC/GG | 59 | 1 |  |  |  |
|  |  | GG | 8 | 1.57(0.696-3.543) |  |  |  |
|  | rs2292832 | TT | 28 | 1 |  |  |  |
|  |  | CT | 34 | 0.577(0.297-1.119) |  |  |  |
|  |  | CC | 5 | 0.183(0.043-0.778) |  |  |  |
|  |  | CT/CC | 39 | 0.497(0.26-0.947) |  |  |  |
|  |  | TT/CT | 62 | 1 |  |  |  |
|  |  | CC | 5 | 0.244(0.058-1.023) |  |  |  |
|  | rs11614913 | TT | 19 | 1 |  |  |  |
|  |  | CT | 33 | 1.447(0.758-2.76) |  |  |  |
|  |  | CC | 15 | 0.987(0.43-2.266) |  |  |  |
|  |  | CT/CC | 48 | 1.297(0.699-2.467) |  |  |  |
|  |  | TT/CT | 52 | 1 |  |  |  |
|  |  | CC | 15 | 0.785(0.384-1.605) |  |  |  |
|  | rs3746444 | AA | 46 | 1 |  |  |  |
|  |  | AG | 21 | 0.652(0.361-1.178) |  |  |  |
| Wang(2012) | rs2910164 | GG | 78 |  |  |  | 1 |
|  |  | CG/CC | 121 |  |  |  | 0.58(0.36-0.94) |
| Yoon(2012) | rs2910164 | CC | 177 |  | 1 |  |  |
|  |  | CG | 159 |  | 0.52(0.31-0.89) |  |  |
|  |  | GG | 51 |  | 0.53(0.24-1.13) |  |  |
|  |  | CG/GG | 210 |  | 0.52(0.32-0.85) |  |  |
|  | rs11614913 | TT | 99 |  | 1 |  |  |
|  |  | CT | 186 |  | 0.66(0.39-1.14) |  |  |
|  |  | CC | 101 |  | 0.67(0.35-1.26) |  |  |
|  |  | CT/CC | 287 |  | 0.67(0.4-1.1) |  |  |
| Tu(2012) | rs2292832 | CC/CT | 149 | 1 |  |  |  |
|  |  | TT | 124 | 1.66(1.05-2.6) |  |  |  |
| Chae(2013) | rs2910164 | GG/CG | 243 |  | 1 | 1 |  |
|  |  | CC | 156 |  | 2.120(1.257-3.574) | 2.349(1.257-4.390) |  |
| Hong(2013) | rs2292832 | TT | 169 | 1 |  | 1 |  |
|  |  | CT | 145 | 0.66(0.46-0.94) |  | 0.66(0.48-0.9) |  |
|  |  | CC | 42 | 0.65(0.35-1.2) |  | 0.59(0.35-1.01) |  |
|  |  | CT/CC | 187 | 0.66(0.47-0.92) |  | 0.64(0.48-0.87) |  |
|  | rs2910164 | CC | 143 | 1 |  | 1 |  |
|  |  | CG | 168 | 0.88(0.61-1.25) |  | 0.94(0.68-1.29) |  |
|  |  | GG | 51 | 0.65(0.36-1.18) |  | 0.74(0.45-1.22) |  |
|  |  | CC/CG | 311 | 1 |  | 1 |  |
|  |  | GG | 51 | 0.7(0.4-1.22) |  | 0.76(0.48-1.23) |  |
|  | rs11614913 | CC | 105 | 1 |  | 1 |  |
|  |  | CT | 181 | 0.67(0.46-0.98) |  | 0.68(0.49-0.95) |  |
|  |  | TT | 74 | 0.77(0.48-1.23) |  | 0.61(0.39-0.93) |  |
|  |  | CT/TT | 255 | 0.7(0.48-0.99) |  | 0.66(0.48-0.90) |  |
|  | rs3746444 | AA | 244 | 1 |  | 1 |  |
|  |  | AG | 98 | 1.02(0.7-1.49) |  | 0.98(0.7-1.38) |  |
|  |  | GG | 17 | 0.61(0.22-1.68) |  | 0.76(0.33-1.75) |  |
|  |  | AA/AG | 342 | 1 |  | 1 |  |
|  |  | GG | 17 | 0.61(0.22-1.66) |  | 0.77(0.34(0.53)) |  |
| Ahn(2013) | rs2910164 | CC | 159 | 1 |  |  |  |
|  |  | CG | 231 | 0.7(0.3-1.4) |  |  |  |
|  |  | GG | 71 | 0.5(0.2-1.8) |  |  |  |
|  |  | CG/GG | 302 | 0.4(0.2-1.1) |  |  |  |
|  | rs2292832 | TT | 241 | 1 |  |  |  |
|  |  | TC | 176 | 0.8(0.4-1.8) |  |  |  |
|  |  | CC | 44 | 1.5(0.6-4.1) |  |  |  |
|  |  | CT/TT | 417 | 1 |  |  |  |
|  |  | CC | 44 | 1.2(0.6-2.5) |  |  |  |
|  | rs11614913 | TT | 119 | 1 |  |  |  |
|  |  | TC | 242 | 1.3(0.2-3.2) |  |  |  |
|  |  | CC | 100 | 1.6(0.6-4.1) |  |  |  |
|  |  | CC/CT | 342 | 1 |  |  |  |
|  |  | TT | 119 | 1.6(0.7-3.7) |  |  |  |
|  | rs3746444 | AA | 323 | 1 |  |  |  |
|  |  | AG | 123 | 2.1(0.9-4.4) |  |  |  |
|  |  | GG | 15 | 1.6(0.4-6.9) |  |  |  |
|  |  | AG/GG | 138 | 1 |  |  |  |
|  |  | AA | 323 | 1.5(0.7-3.1) |  |  |  |
| Umar(2013) | rs11614913 | CC | 146 | 1 |  |  |  |
|  |  | CT | 121 | 0.9(0.59-1.36) |  |  |  |
|  |  | TT | 22 | 1.04(0.41-2.61) |  |  |  |
|  | rs2910164 | GG | 163 | 1 |  |  |  |
|  |  | GC | 102 | 0.84(0.56-1.28) |  |  |  |
|  |  | CC | 24 | 0.45(0.17-1.17) |  |  |  |
|  | s3746444 | TT | 155 | 1 |  |  |  |
|  |  | TC | 122 | 0.89(0.59-1.35) |  |  |  |
|  |  | CC | 12 | 0.83(0.37-1.87) |  |  |  |
| Guan(2013) | rs2910164 | CG/CC | 115 | 1 |  | 1 |  |
|  |  | GG | 166 | 0.5(0.3-1) |  | 0.3(0.1-0.8) |  |
|  | rs11614913 | CC | 87 | 1 |  | 1 |  |
|  |  | CT/TT | 194 | 0.4(0.2-0.8) |  | 0.3(0.1-0.7) |  |
|  | rs2292832 | CT/TT | 131 | 1 |  | 1 |  |
|  |  | CC | 150 | 0.6(0.3-1.2) |  | 1.3(0.6-3.2) |  |
|  | rs3746444 | CT/CC | 121 | 1 |  | 1 |  |
|  |  | TT | 160 | 1(0.5-2) |  | 0.8(0.3-1.9) |  |
| Liu(2013) | rs11614913 | TT | 104 | 1 |  |  |  |
|  |  | CT/CC | 211 | 1.76(1.11-2.79) |  |  |  |
| Navarro(2013) | rs11614913 | CC | 61 |  |  | 1 |  |
|  |  | CT/TT | 79 |  |  | 0.4(0.1-1.1) |  |
| Wang(2013) | rs11614913 | CT/TT | 773 | 1 |  |  |  |
|  |  | CC | 167 | 0.72(0.55-0.95) |  |  |  |
| Zhang(2013) | rs2292832 | CC | 6 | 1 |  |  |  |
|  |  | CT | 53 | 0.5(0.17-1.49) |  |  |  |
|  |  | TT | 39 | 0.58(0.20-1.69) |  |  |  |
| Kim(2014) | rs11614913 | TT | 364 | 1 |  |  |  |
|  |  | CT/CC | 718 | 0.97(0.88-1.06) |  |  |  |
|  |  | TT/CT | 356 | 1 |  |  |  |
|  |  | CC | 256 | 0.9(0.431-1.88) |  |  |  |
| Wu(2014) | rs2910164 | GG | 119 | 1 |  |  |  |
|  |  | CG | 181 | 0.887(0.648-1.214) |  |  |  |
|  |  | CC | 75 | 0.894(0.699-1.144) |  |  |  |
|  |  | CG+CC | 256 | 1.059(0.43-1.189) |  |  |  |
|  |  | CG+CC CC | 256  75 | 0.949(0.720-1.252) |  |  |  |
|  | rs11614913 | CC | 85 | 1 |  |  |  |
|  |  | CT | 184 | 1.141(0.864-1.507) |  |  |  |
|  |  | TT | 106 | 1.584(1.16-2.162) |  |  |  |
|  |  | CT+TT | 290 | 1.268(0.976-1.648) |  |  |  |
|  |  | CC+CT  TT | 290  106 | 1  0.691(0.541-0.882) |  |  |  |
