# Supplementary material for: Prognostic Role of Common MicroRNA Polymorphisms in Cancers: Evidence from a Meta-Analysis
Source: PLoS One. 2014 Oct 22;9(10):e106799. doi: 10.1371/journal.pone.0106799 (PMC4206268; doi:10.1371/journal.pone.0106799)
Supplement: Table S3 — The association of mirna polymorphisms with DFS and RFS. (DOC) [file pone.0106799.s003.doc]

Table S3 The association of mirna polymorphisms with DFS and RFS

| Mir | Genotypes | DFS | Genotypes | RFS |
| --- | --- | --- | --- | --- |
| Mir-146a | CG+GG | 1 | GG+CG | 1 |
|  | GG | **0.649(042-0.996)*** | CC | 0.669(0.371-1.205) |
| Mir-19a62 | CC | 1 | TT | 1 |
|  | CT+TT | 0.887(0.417-3.19) | CT | **0.675(0.485-0.94)*** |
|  |  |  | CC+CT | **0.687(0.504-0.936)*** |

*P<0.05, DFS: disease-free survival, RFS: recurrence-free survival
