# Supplementary material for: Prognostic Role of Common MicroRNA Polymorphisms in Cancers: Evidence from a Meta-Analysis
Source: PLoS One. 2014 Oct 22;9(10):e106799. doi: 10.1371/journal.pone.0106799 (PMC4206268; doi:10.1371/journal.pone.0106799)
Supplement: File S1 — The seven excluded articles and the reasons. (DOC) [file pone.0106799.s004.doc]

The seven excluded articles and the reasons.

1 Okubo M, Tahara T, Shibata T, Yamashita H, Nakamura M, Yoshioka D, Yonemura J, Kamiya Y, Ishizuka T, Nakagawa Y, et al: Association between common genetic variants in pre-microRNAs and the clinicopathological characteristics and survival of gastric cancer patients. Experimental and therapeutic medicine 2010, 1:1035-1040.

2. Dikeakos P, Theodoropoulos G, Rizos S, Tzanakis N, Zografos G, Gazouli M: Association of the miR-146aC>G, miR-149T>C, and miR-196a2T>C polymorphisms with gastric cancer risk and survival in the Greek population. Molecular biology reports 2014, 41:1075-1080.

3. Chae YS, Kim JG, Kang BW, Lee SJ, Lee YJ, Park JS, Choi GS, Lee WK, Jeon HS: Functional Polymorphism in the MicroRNA-367 Binding Site as a Prognostic Factor for Colonic Cancer. 2013, Anticancer Research:513-520.

Resons: The three articles above lack of data.

1. Zheng J, Deng J, Xiao M, Yang L, Zhang L, You Y, Hu M, Li N, Wu H, Li W, et al: A sequence polymorphism in miR-608 predicts recurrence after radiotherapy for nasopharyngeal carcinoma. Cancer research 2013, 73:5151-5162.

Resons: The article discussed the association between polymorphisms and time to recurrence not overall survival.

1. Xing J, Wan S, Zhou F, Qu F, Li B, Myers RE, Fu X, Palazzo JP, He X, Chen Z, Yang H: Genetic polymorphisms in pre-microRNA genes as prognostic markers of colorectal cancer. cancer epidemiol biomarkers prev 2012, 21:217-227.

2. Lin J, Horikawa Y, Tamboli P, Clague J, Wood CG, Wu X: Genetic variations in microRNA-related genes are associated with survival and recurrence in patients with renal cell carcinoma. Carcinogenesis 2010, 31:1805-1812.

3. Stenholm L, Stoehlmacher-Williams J, Al-Batran SE, Heussen N, Akin S, Pauligk C, Lehmann S, Senff T, Hofheinz RD, Ehninger G, et al: Prognostic role of microRNA polymorphisms in advanced gastric cancer: a translational study of the Arbeitsgemeinschaft Internistische Onkologie (AIO). Annals of oncology : official journal of the European Society for Medical Oncology / ESMO 2013, 24:2581-2588.

Reasons: The three articles above lack of precise genotypes.
